# A paucity of strategies for developing health literate organisations: A systematic review

**DOI:** 10.1371/journal.pone.0195018

**Published:** 2018-04-11

**Authors:** Jane E. Lloyd, Hyun J. Song, Sarah M. Dennis, Nicola Dunbar, Elizabeth Harris, Mark F. Harris

**Affiliations:** 1 Health Equity Research and Development Unit, Sydney Local Health District, Sydney, NSW, Australia; 2 Centre for Primary Health Care and Equity, University of New South Wales, Sydney, NSW, Australia; 3 Clinical and Rehabilitation Sciences, Faculty of Health Sciences, University of Sydney, Lidcombe, NSW, Australia; 4 Australian Commission on Safety and Quality in Health Care, Sydney, NSW, Australia; Brown University, UNITED STATES

## Abstract

**Introduction:**

People with low health literacy are more likely to delay seeking care and experience adverse outcomes. While health literacy is the product of individuals’ capacities, it is also affected by the complexities of the health care system. System-level changes are needed to align health care demands better with the public’s skills and abilities. We aimed to identify the evidence base for effective strategies for creating health literate organisations.

**Methods:**

A systematic review and narrative synthesis of empirical studies was performed. Medline, Embase, PsychInfo and CINHAL databases were searched for empirical studies from OECD countries published from 2008 onwards, focusing on health literacy interventions at the organisational level. Analysis of the findings was informed by the National Academies’ five-dimensional framework for the attributes of a health literate organisation, which include: organisational commitment, accessible education and technology infrastructure, augmented workforce, embedded policies and practices, and effective bidirectional communication.

**Results:**

The title and abstract of 867 records were screened according to the selection criteria, leading to full text review of 125 articles. Seven studies were identified in the peer review literature. Adapting health literacy guidelines and tools was the most common approach to addressing organisational health literacy.

**Conclusion:**

While the use of health literacy tools proved important for raising awareness of health literacy issues within organisations, these tools were insufficient for generating the organisational changes necessary to improve organisational health literacy.

## Introduction

### Why is health literacy important to the health of populations?

Over the last twenty years the focus has shifted away from treating acute illness and towards establishing systems and processes to support the ongoing management of chronic conditions [1]. This shift stems from the fact that people are increasingly living with lifestyle-related chronic (ongoing) diseases, health conditions, health risks and disability [2]. The growing emphasis on chronic conditions has led to an increasing reliance on self-management, putting greater onus on patients to manage their own health care. Increasingly, complex care is being transferred out of the hospital into the community and is expected to be managed by patients and their carers [1].

The growing complexity of health care is a challenge to the vast majority of patients. The patient-centered movement requires all patients to be health literate if they are to be partners in their own care [3]. Globally, health literacy is recognised as a pillar for improving the health of populations. In 2014, Australia identified building health literacy as a national priority with the publication of the National Health Literacy Statement [2]. Health literacy can be defined as the knowledge, skills, confidence and networks that are necessary for staying healthy, accessing preventive screening, deciding on treatment options, self-management and effective communication [4–6].

Health literacy is also an important factor to address in order to improve equitable access to health care. The European Health Literacy survey measured health literacy in eight countries (n = 8000) and found 47% percent of respondents had insufficient or problematic health literacy. The distribution of health literacy levels differed substantially across countries (29–62%). Disadvantaged populations had higher proportions of people with low health literacy [7]. A separate survey in Australia conducted in 2006, found sixty percent of Australians have health literacy below what is necessary to access appropriate health care [8]. Low health literacy disproportionally affects disadvantaged Australians which, in turn, impacts on mortality and burden of disease as well as health service use and costs [9–11].

### The role of health care organisations in supporting people with low health literacy to access and benefit from health care

While health literacy is partly the product of individuals’ capacities, it is also affected by the demands and complexities of managing chronic diseases and the navigating the health care system [5, 12]. System and organisational changes are needed to align health care demands better with the public’s skills and abilities [13].

Organisations within the health care system, such as hospitals, pharmacies and general practices, can reduce the level of health literacy required to access health care by making services, education and information more appropriate for people with low health literacy. Organisations can also seek to develop individual, family and community knowledge, skills and capabilities. There are some efforts towards building individual competencies and capacity, for example self-management courses; however, there has been little progress towards reducing the complexity of the health care system and health information. Patients are often awash with health information and there is evidence that services are becoming increasingly difficult to navigate [14, 15].

**Fig 1:**
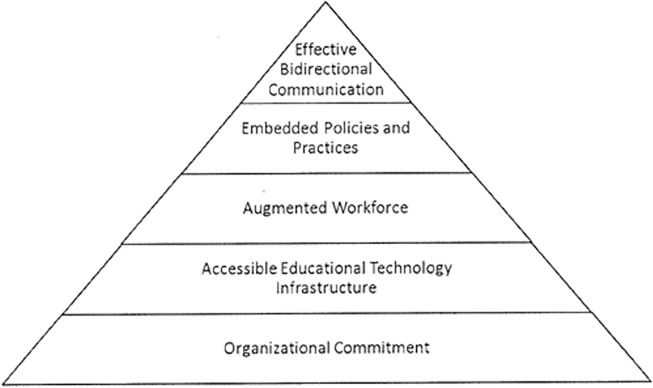
Health and Medicine Division’s framework for the health literate health care organisation, developed by Schillinger and Keeler [13].

Health care organisations that make it easier for people to navigate, understand and use information and services have been described as health literate organisations [16]. The framework for a health literate organisation (depicted in “Fig 1”) describes the following five attributes of a health literate organisation: organisational commitment; accessible education and technology infrastructure; augmented workforce; embedded policies and practices; and effective bidirectional communication [17]. In health literate organisations all staff–clinicians, administrators, managers and leaders–prioritise health literacy as part of their work and understand that ‘no matter how high a consumer’s level of health literacy is, stress and anxiety affect their ability to understand and remember new information’ [18] p.2.

Focusing on improving organisational health literacy steers away from more traditional health literacy interventions where the focus is heavily on individuals and their capacity, instead putting the onus on health services to systematically build their capacity to create a more responsive, accessible, and comprehensible setting for patients [19]. The Australian Commission on Safety and Quality in Health Care define organisational health literacy interventions as those which target the infrastructure, policies, processes, materials, people and relationships that make up the health system and have an impact on the way in which people access, understand, appraise and apply health-related information and services [16]. In 2012, the Commission conducted a stocktake in Australia, by calling for submissions, and found 227 health literacy interventions. According to their analysis the main strategies used to improve health literacy include: providing health information (47%); researching and sharing knowledge about health literacy (21%); improving the skills of individuals (14%); examining or changing the health service environment (7%); training the health workforce (7%); and developing polices and frameworks (4%) [20].

Given the evolving understanding of health literacy and the increasing recognition of organisations’ need to address health literacy, we sought to examine the global evidence base for effective interventions to improve organisational health literacy in the peer-reviewed literature.

Our systematic review aimed to address two questions: (1) What is the evidence base for strategies and interventions being implemented by health care organisations to address organisational health literacy? and (2) What are the barriers and facilitators of adopting or implementing these strategies and the implications for health literacy in Australia?

## Methods

A protocol for the review was developed and discussed with a research advisory group (MH, EH, SD, ND) in order to ensure that the methods and search strategies were exhaustive. The following databases were searched: Medline, Embase, CINAHL, PsycINFO. The date last searched was 10^th^ July 2015.

### Study selection

To retrieve studies, keywords and MeSH terms relating to ‘organisational change’ and ‘health literacy’ were combined with a health literacy focus term (e.g. *Health Literacy/ in Medline) using the Boolean operator *and* (S1 Appendix Keywords and MeSH terms used in database search). From the resulting list, studies were selected for inclusion in the review based on the relevance of their title and abstract. To ensure consistency 10% of the studies were double screened. Eligible studies were included which met the following inclusion criteria: (1) written in English; (2) published between January 2008 and July 2015; (3) research was conducted in an OECD country; (4) was an intervention study, program evaluation, or needs assessment; (5) addressed the health literacy of adults, aged ≥18 years; (6) focused on change at the organisation or system level, and not solely on individual-level interventions; and (7) evaluated interventions in line with the framework for a health literate organisation developed by the Health and Medicine Division (HMD) of the National Academies of Sciences, Engineering, and Medicine (formerly known as the Institute of Medicine) [1]. Further details on the study selection criteria are included in S2 Appendix: Study selection criteria. The timeframe of January 2008 and July 2015 was chosen to capture the four year period before and after the seminal publication on organisational health literacy, which was a report by the Institute of Medicine identifying attributes of a health literate organisation [13].

Studies were excluded if they lacked an evaluative component testing the effectiveness of an intervention or strategy aimed at addressing organisational health literacy. We excluded studies that focused on improving individual health literacy of the patient or family, as well as studies that only measured or described underlying determinants or factors associated with health literacy (e.g. link between education level and health literacy). Studies were also excluded if their primary focus was on the measurement or assessment of health literacy (e.g. validity testing of health literacy screening tools). The HMD’s Framework for Health Literate Organisations served as a general guideline for the inclusion and classification of interventions. However, studies were not necessarily excluded if the organisation-based intervention could not be categorised into one or more of the frameworks.

### Data extraction

Data were extracted on study characteristics, intervention components, intervention context (adopters and implementers, organisational setting and rationale for change), methods and results (outcomes, barriers and facilitators). Two reviewers (JL, HS) independently extracted data on 40–60% of the studies respectively. The aim of the data extraction process was to describe a body of literature, rather than to determine the size of an effect, therefore no risk of bias assessment was made [21].

### Quality assessment

Included studies were reviewed for quality assessment using an adapted version of the Standard Quality Assessment Criteria for Evaluating Primary Research Papers [22]. The ten criteria the quality was assessed against included:

1. Research question sufficiently described
2. Study design evident and appropriate
3. Context for study clear and use of wider body of knowledge
4. Use of a conceptual framework
5. Sampling strategy described, relevant and justified
6. Data collection methods clearly described and systematic
7. Data analysis clearly described and systematic
8. Use of verification procedures to establish credibility
9. Conclusions supported by the results
10. Reflexivity of the account

Two reviewers (JL, HS) independently assessed the quality of the studies. The reviewers then met to compare their results. Differences were resolved by discussing the rationale behind the rating until consensus was reached. The numerical scores were converted to rating of weak (0–4), moderate (5–7), or high (8–10). See Table 1 for details.

**Table 1:** Overview of the included studies.

Author year	Setting	Target	Aim	Intervention	Findings
Blake et al 2010 [31]	Pharmacy–three outpatient pharmacies of an inner-city health system in Atlanta, Georgia	Primarily patients, focussing on at-risk populations; Pharmacists and pharmacy staff	To evaluate the implementation of a health literacy intervention to improve medication adherence and processes of care among patients in an inner-city health system	Three components 1. Automated telephone reminder (ATR) service which allowed patients to trigger refills over the phone. 2. PictureRx, a software that was installed in pharmacies and printed personalised illustrated pill cards for patients to help them understand what pills are used for what purpose and when to take them. 3. Training pharmacists and pharmacy staff in clear and simple communication techniques.	The findings were based on the analysis of qualitative interviews. Patients indicated they were pleased overall and especially with the PictureRx card which was described as useful and helpful. The ATR was easy to understand, calls were received at convenient times, reminded patients to call in their refills, helped avoid a long line at pharmacy, changed their refill behaviour and helped get refills on time. Pharmacists and pharmacy staff indicated they were pleased with the communication training; learned how easily patients can bet confused about medication instructions, became more careful and specific in providing instructions to their patients, avoided medical jargon, reported to now assess patients understanding more carefully and make more of an effort to talk about side effects.
Shoemaker et al 2013 [32]	Eight pharmacies	Pharmacies as an organisation, to examine their organisational culture, capacity, values and other factors.	To understand the facilitators and barriers to the adoption and implementation of the Agency for Healthcare Research and Quality (AHRQ) health literacy tools, especially to assess the health literacy practices of pharmacies.	A comparative, multi-case study of eight pharmacies, guided by an adaption of Rogers’s Diffusion of Innovations model. The four AHRQ health literacy tools for pharmacies includes an assessment tool; a guide on creating a pill card, a staff training guide, a guide on creating automated telephone refill reminders.	The findings were based on analysis of interviews, site visit observations and a review of documents. The analysis indicated that factors important to pharmacies’ decision to adopt the health literacy tools included awareness of health literacy, a culture of innovation, a change champion, the relative advantage and compatibility of the tools. Facilitators to implementation of the tools included buy-in from leadership, qualified staff, college-affiliated change champions, the adaptability and organisation of the tool and support. Barriers to implementation were limited leadership buy-in, prioritisation of other activities, lack of qualified staff and tool complexity.
Cawthon et al 2014 [33]	University Medical Centre which includes a 658-bed hospital, outpatient facilities and three primary care practices	Nursing staff	To implement the Brief Health Literacy Screen, in a large academic medical centre	A four-part strategy was used to implement the Brief Health Literacy Screening (BHLS) tool. The BHLS is a 3-item tool assessing patient’s ability to read and understand medical information, as well as fill out forms. The implementation strategy included: the selection of tool suitable for nursing workflow; garnering key nurse leaders’ support and participation; providing education and training on the use of the tool; electronic health record integration; and ongoing evaluation and feedback. In the outpatient setting the BHLS was reassessed for each patient after 12 months. In the in-patient setting the BHLS was assessed at each hospitalisation. Implementation was based on a quality improvement framework with a focus on acceptability, adoption, appropriateness, feasibility, fidelity and sustainability.	The findings were based on querying the Enterprise Data Warehouse which contained data from the electronic health record; direct observations; and focus groups, interviews and process recordings. The completion rate in the hospital over a five-month period was 91.8%. For outpatient clinics, the completion rate was 66.6%. The results indicate that it is feasible to incorporate health literacy screening into clinical assessment and electronic health records. The next challenge will be to evaluate the association of health literacy with processes and outcomes of care.
Johnson 2014 [14]	Rural hospital in South Australia	Organisations health literacy from the patient perspective	To identify how a rural health service could improve their organisational health literacy, the barriers and enablers patients face when they physically navigate their way to and around the health service.	A case study of the health literacy demands placed on consumers when attending a health service. The First Impressions and Walking Interview tool was used to assist health services to begin to consider some of the characteristics of their workplace that help or hinder a consumer’s ability to physically navigate their way to and bout the health service. One consumer was the reviewer for the phone call activity and the other consumer was the reviewer for the web page. Both consumers undertook the walking interview as observers and provided feedback on their journey and suggested improvements to navigation of the health service. The Safety and Quality Coordinator was the guide for the walking interview.	The First Impressions Activities did identify the barriers and enablers that patients face when they need to access and navigate the health service. Consumers perspectives obtained via the First Impressions Activities provided the hospital management with clear direction on how access and navigation might be improved, and the health literacy demands on consumers might be reduced. There was no discussion about what had changed and what was going to be done differently as a result of the needs assessment.
Groene et al. 2011 [23]	Ten hospitals in Catalonia, Spain	The focus of attention for the assessment of written and oral communication were patients that undergo cataract surgery in outpatient departments in nine hospitals (one hospital did not perform this surgery).	1. Identify the factors that hinder or support the ability of people to make their way to and within a hospital or health care centre 2. Pilot the assessment of literacy issues within hospitals settings	The intervention included an assessment of the health literacy environment of 10 hospitals in Catalonia, Spain. Standardised rating tools were developed and used for the evaluation of the hospitals navigability, and to assess the reading ability of written communication. A patient survey was conducted to evaluate patient perceptions of written and oral communication.	The tools identified barriers and facilitators to health literacy in the hospital setting including: • Navigation: insecurity and confusion in finding one’s way throughout health care facilities • Written communication: extensive use of scientific language in selected health education materials and informed consent documents that is inappropriate for the general public (education level below university degree). • Patients’ perception of written and oral communication: oral communication rated high, but variations between hospitals exist. The assessment of health literacy environment is an important step in identifying improvement opportunities, however on its own is insufficient in generating change.
Smith et al. 2010 [24]	A stroke unit in a rehabilitation hospital and a senior independent living facility in the United States	Focuses on the role of occupational therapist in addressing health literacy via written information and the navigability of health services.	To present a review of the accessibility of a rehabilitation centre and an independent living facility with regard to navigation of the facility, understandability of written and oral communication, use of technology, and implementation of policies and procedures within these facilities.	Two reviewers were involved in the review of each faculty, which allowed for comparison of data between reviewers within the site. The reviewers met with the administrators of each facility to identify areas to be assessed and were given a tour of the facility	The review identified organisational health literacy strengths and weaknesses. Strengths included the use of plain language and an engaging style with during interactions. The weakness was the lack of multilingual staff and translation services. At the rehabilitation centre staff did not check for understanding. While telephones, televisions and computers were available they were used minimally. New staff are expected to participate in orientation programs, but health literacy is not discussed. The authors argue that the American Occupational Therapy Association write a position paper support concepts of health literacy. The paper did not identify specific roles of occupational therapists above and beyond other health professionals.
Weaver et al. 2012 [25]	Three primary health care clinics in rural Missouri, United States	Health service administrators, clinical and non-clinical support staff and patients were involved in measuring the health literacy policies and practices in three rural primary health care services.	To prepare a health literacy policy action plan with special attention to organisational factors and to then implement and evaluate the policy action plan. A needs assessment was carried out in three clinic locations to identify strengths and weaknesses in existing health literacy practices and organisational factors that would facilitate and impede efforts to enhance health literacy practices.	‘Rudd and Anderson’ was the organising framework for the needs assessment. Three customised instruments were used to assess the domains of organisational health literacy: An observational assessment Key informant interview guides for clinic staff Key informant interview guide for patients.	The customised needs assessment was seen as contributing to an ongoing collaborative process to implement organisational changes that address health literacy needs. Agreed actions included: 1. Establishing tools and processes for systematically reviewing and standardising patient education materials. 2. Staff meetings were identified as opportunities to discuss health literacy and organisational changes during and after the project. 3. The team agreed to develop a form for patients to use to record the physician discussion, the outcome of the visit, and the recommended treatments.

## Results

**Fig 2:**
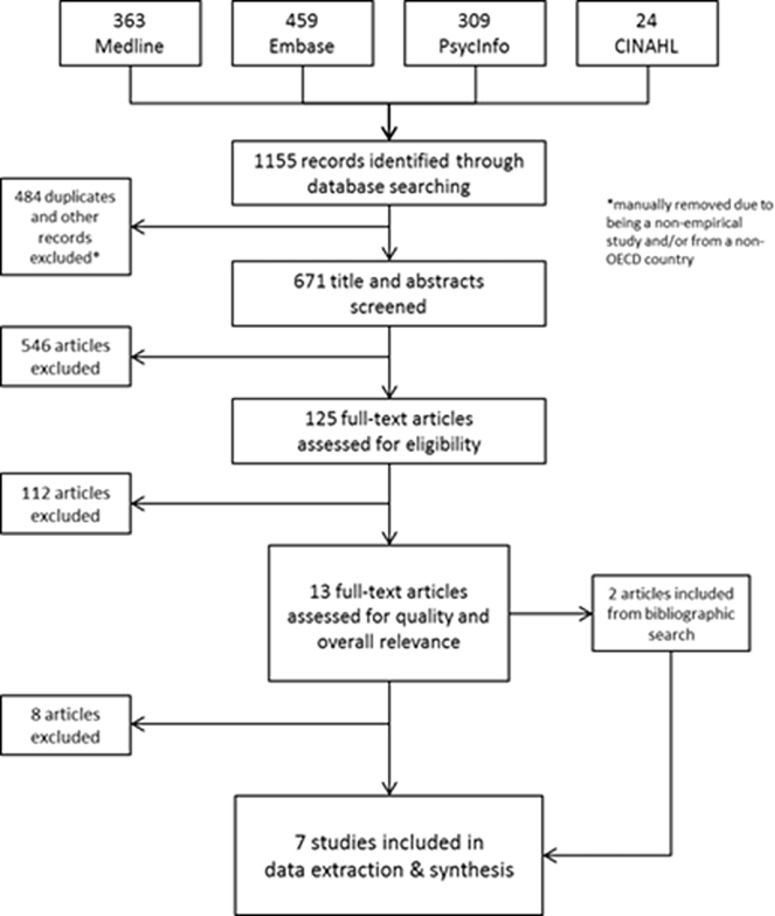
PRISMA flowchart detailing flow of studies through the review.

The database search identified 1155 records. After the removal of duplicates, non-empirical studies and studies from non-OECD countries 671 papers remained. Many studies were excluded due to their lack of an organisational focus and on the basis of evaluating interventions that were designed and implemented outside of the health sector (e.g. health literacy training program as part of higher education curriculum). One hundred and twenty-five papers were screened on full text, and thirteen studies were initially included on full text, but eight were removed upon further review. Two studies were added from bibliographic search. In total, seven studies were included for data extraction. Four of the seven studies were assessed to be of moderate quality and three of the studies were assessed to be of high quality. “Fig 2” (included at the end of the manuscript) describes the flow of studies through searching and screening for inclusion using the Preferred Reporting Items for Systematic Reviews and Meta-Analysis (PRISMA) flowchart.

### Characteristics of included studies

The seven studies were published between 2010 and 2014 and were undertaken in United States, Spain, and Australia. The Groene and Rudd study was the largest in scope, having been conducted across ten hospitals in Spain [23] whereas the Johnson study was conducted in one small rural hospital in Australia [14].

Three of the studies highlighted the health literacy experience of vulnerable populations. Two studies placed their focus on rural communities–one in a small health service in Australia [14] and one in three primary health care clinics in the US. Of the two pharmacy-based studies, Blake et al. targeted underserved patients in an inner-city health system and in their discussion, they emphasised the value of using a tool that can be tailored to their patients’ needs.

Two studies focused on a diverse patient population in terms of socioeconomic status and educational level but did not comment on equity issues in the finding and discussion sections of their papers. Both the Smith and Groene studies concerned elderly patient populations–Smith et al. conducted half of its study within a senior independent living facility [24], and Groene and Rudd [23] examined primarily elderly patients in outpatient cataract surgery units.

### Types of interventions

Three distinct intervention approaches were identified from the seven included studies, which included environmental scans, pharmacy health literacy tools, and health literacy screening. The results are discussed below according to each of these approaches. For a full description of the included studies, see S3 Appendix: Methods used in the included studies.

#### The environmental scans

Four studies conducted an environmental scan of the health care organisation to ascertain organisational responsiveness to patients’ health literacy needs [14, 23–25]. These four studies either used Harvard University’s *Health Literacy Environment of Hospitals and Health Centers* toolkit, which contains a set of review tools for assessing and analysing literacy-related barriers to healthcare access and navigation [26] or the *First Impressions and A Walking Interview* packet which is based on the former toolkit [27]. The toolkit includes a series of preliminary activities and self-assessment questionnaires to evaluate five different aspects of the health literacy environment: navigation, written/print communication, oral communication, technology, and organisational policies [26].

The adaption, rather than adoption, of the environmental scan was common, as was the use of further tools to inform organisational health literacy performance. The Groene and Weaver studies [23, 25] combined the use of the Harvard toolkit with other existing resources including the Agency for Health Research and Quality’s *Health Literacy Universal Precautions Toolkit* [28], the Joint Commission’s set of organisational health literacy recommendations [29], and Fry’s Readability Graph [30]. While Smith, Groene and Weaver adapted the same tool to conduct their environmental scan, they used different data collection methods.

Johnson opted to use the *First Impressions and A Walking Interview* packet [27] as a way to “engage with consumers to examine the aspects of health literacy in navigating their way to and around a health service with ‘fresh eyes’”. This packet assesses patient experience when making first contact with the clinic or hospital (by telephone and web access), and in physically navigating the building. The patient feedback highlights the strengths and weaknesses of the organisational health literacy and therefore how it might be improved from the patients’ perspective.

All four studies reviewed the navigability of the health service, including signage, assistance from staff, and ease of locating certain areas of the building. Smith, Groene and Weaver studies assessed both print (e.g. readability and clarity of written material) and oral communication (e.g. the use of clear, plain language from staff). Only Smith and Weaver performed an evaluation of technology (e.g. availability of televisions, telephones, and computers to patients in the clinic) as well as the policies and protocols of the health care organisation. The Johnson study did not assess any health literacy aspect other than navigation, since it used the First Impressions toolkit, which mainly analysed initial access and navigability issues.

The most common finding from the environmental scan was the need to improve navigation within the hospital. It is difficult for patients to find their way around the health care facilities. This was attributed to problems with signage such as inconsistent terminology, or over use of scientific language, or that the signage was missing or obscured [14, 23, 24].

The Weaver study found that there was a low awareness of health literacy within the organisations protocols, inter-staff communication and patient communication [25]. But this was countered against high employee morale and patient satisfaction.

The environmental scans were conducted in a diverse range of setting. These settings included a stroke unit in a rehabilitation facility and a senior independent living facility [24], three primary health care clinics in rural Missouri [25], a small rural hospital in South Australia [14] and patients who had cataract surgery and attended outpatient departments in ten hospitals in Catalonia, Spain [23].

The successful application of the Health Literacy Environmental Review in rural and urban locations in three countries, within both tertiary and primary health care settings and across different disciplines such as ophthalmology and occupational therapy, demonstrate the flexibility and adaptability of the tool. The assessments provided feedback on the health literacy of the organisations. This is important to note but does not in and of itself lead to a change in practice.

#### Pharmacy health literacy tools

Both of the pharmacy studies evaluated the adoption of the AHRQ health literacy tools for pharmacies. The tools comprise an assessment tool; a guide on creating a pill card, a staff training guide, a guide on creating automated telephone refill reminders. Blake et al., piloted and evaluated the uptake of the intervention in three pharmacies. The other study, Shoemaker, conducted a case study across eight pharmacies to assess the facilitators and barriers to the adoption and implementation of the AHRQ health literacy tools.

Blake evaluated patient and pharmacy staff satisfaction with the use of those tools [31]. Blake’s findings were based on the analysis of qualitative interviews. Patients indicated they were pleased overall and especially with the PictureRx card which was described as useful and helpful. The automated telephone reminder service was easy to understand, calls were received at convenient times, reminded patients to call in their refills, helped avoid a long line at pharmacy, changed their refill behaviour and helped get refills on time. Pharmacists and pharmacy staff indicated they were pleased with the communication training; learned how easily patients can bet confused about medication instructions, became more careful and specific in providing instructions to their patients, avoided medical jargon, reported to now assess patients understanding more carefully and make more of an effort to talk about side effects [31].

Shoemaker adapted a Diffusions of Innovation Model to identify the barriers and enablers to adopting and implementing the AHRQ health literacy tools across eight pharmacies [32]. The results suggest that the primary effect of using the assessment tool (AHRQ) was an increased awareness of health literacy among pharmacy staff. However, many of the pharmacists in the study struggled to define a course of action based on the assessment results because of their limited knowledge of how to implement health literacy strategies in practice, and found that the tool did not offer adequate guidance on how to translate results into action [32].

#### Health literacy screening

The Cawthon study incorporated the Brief Health Literacy Screening tool (BHLS) into the electronic record at a large academic medical centre in the US [33]. This service incorporated adult hospital units, the emergency department and three primary care practices. The incorporation of the tool enabled all new patients to be screened for health literacy on admission. The BHLS is a 3-item tool assessing patient’s ability to read and understand medical information, as well as fill out forms. The implementation strategy included: the selection of tool suitable for nursing workflow; garnering key nurse leaders’ support and participation; providing education and training on the use of the tool; electronic health record integration; and ongoing evaluation and feedback. The uptake was 91.8% at three months suggesting it is feasible to incorporate health literacy screening into clinical assessment and electronic health records. However the authors did not describe if and how the health literacy information was used by clinicians as part of their care. The tool was successful in recording health literacy status but insufficient for ensuring that patient care was tailored to their health literacy status.

### What were the barriers and facilitators of change across the three types of interventions?

The Shoemaker and the Blake studies are the only studies which explicitly report on the change process that may be required in order to build organisational health literacy. The Shoemaker study identified factors such as a culture of innovation, a change champion, awareness of health literacy as important starting points which enable organisations to adopt health literacy tools. The authors also examined barriers and facilitators to implementation. They found that the adoption and implementation of health literacy tools was not successful in pharmacies that had one or more of the following characteristics: competing initiatives or limited staff availability; decided that the advantage of the intervention was worth the investment; perceived the tool to be too long or complex; limited buy-in from leadership; prioritisation of patient care over implementing tool; and a lack of qualified staff and supervision.

The pharmacists in the Blake study reported that counselling was facilitated by using the PictureRx tool and that patients liked the reminder calls and the illustrated medication schedule which they were able to take home with them. In their conclusion the authors comment on components of successful health literacy interventions. These include the need to adopt tools that are easy to comprehend, are accessible and able to personalised. A key component of the Cawthorn study’s implementation strategy was to select a tool suitable for nursing workflow. Therefore, three studies suggest that adopting a tool that is not too complex, can be tailored, and is appropriate to the setting is an important facilitator of change.

The design of the Cawthorn study factored in the important facilitators of implementing tools. Namely gaining leadership support, ensuring the tool was suitable and acceptable to the workforce and using a quality improvement framework to support and monitor the implementation process.

The barriers and facilitators of change are universal and not specific to organisational health literacy. Leadership buy-in and support is widely recognised as important to support implementation. This suggests that if we are to improve organisational health literacy we need to understand the concept and practice of health literacy in addition to understanding how change works and benefits are sustained.

## Discussion

### Weak evidence base

Despite a large and increasing literature on health literacy generally [34], and individual health literacy interventions in particular [35], the evidence base for strategies and interventions to address organisational health literacy is weak. The seven studies included in this review adapted health literacy tools into their health service. Four of these studies conducted environmental scans, which are similar to needs assessments, to identify where organisational health literacy needed to be improved. Two adapted the AHRQ health literacy tools for pharmacies and one incorporated the BHLS into the electronic record at a large academic medical centre in the US.

### Where progress has been made

Despite the lack of evidence for effective organisational health literacy interventions some progress has been made. This systematic literature review found that organisational health literacy guidelines and tools were the common approach to addressing organisational health literacy. The use of these tools and guidelines were effective in raising awareness of the organisational strengths and weaknesses relating to organisational health literacy. They had high face validity as they were used in various settings and applied in diverse ways using different data collection methods and analysis. The inherent flexibility in adaptation of the tools may be both a strength and a weakness. The broad adaption of the tool may be a strength because of its reach, and a weakness because the quality of the application may vary and the findings cannot be compared against one another.

However, despite the tools providing feedback on current performance, none of the three tools resulted in changes in practice. In the case of the environmental scans and the BHLS this is because the tools are intended to provide feedback and information on performance. While they helped to reveal the health literacy level of organisations and provided an opportunity for reflection, in and of themselves these environmental scans did not change practice. In other words, the tools highlight the problems but do not necessarily provide solutions. For instance, the BHLS was successful in recording health literacy status but was insufficient for ensuring that patient care was tailored to their health literacy status.

The AHRQ tool kit included assessment and intervention components such as the guides on creating a pill card for patients and on creating automated telephone refill reminders. The Blake study piloted the intervention and the authors measured pharmacist and patient satisfaction. The Shoemaker study examined the barriers and facilitators to the uptake of the tool. There was evidence of satisfaction with the use of the AHRQ tools but not for change in patients’ access to quality care or improved health literacy. The results of the Shoemaker study suggest that the primary effect of using the assessment tool (AHRQ) was an increased awareness of health literacy among pharmacy staff. However, many of the pharmacists in the study struggled to define a course of action based on the assessment results because of their limited knowledge of health literacy best practices and found that the tool did not offer adequate guidance on how to translate results into action.

### Using tools and guidelines to address complex and universal problems

This systematic literature found that instead of organisational health literacy strategies and interventions, tools and guidelines were identified as the main mechanisms for change. The use of guidelines and tools is common practice in health services research, however their effectiveness is limited. For example a systematic literature review found that clinical practice guidelines only lead to a 10% improvement in outcomes of care [36].

Using tools and guidelines to address complex and universal (as opposed to disease-specific) problems is supported by the literature. Most notably a systematic review assessed existing measures that assess one or more of the ten attributes of organisational health literacy [37]. They found 68 tools, 12 which addressed five or more of the ten attributes of health literate organisations; 27 tools that addressed between two and four attributes; 29 tools that addressed one attribute of health literate organisations, most commonly interpersonal communication. Twenty six of the 29 tools focussed on interpersonal communication. The authors argue that having a broad array to tools available enables organisation to assess their organisational health literacy and to track their progress [37].

The use of tools, instruments and frameworks to respond to complex problems is not specific to organisational health literacy, a similar response can be found in other areas such as improving the cultural competence of health services [38, 39]. For example Betancourt presents a framework for addressing racial disparities in health and health care that recommends minority recruitment into the health professions, development of interpret services and language-appropriate health education materials, and provider education on cross cultural issues [38]. This emphasises what needs to change but not *how* to go about it.

Many of the discussion papers on improving organisational health literacy highlight what might need to change, however they do not include details for *how* to generate change. Paasche-Orlow et al promote three principles health care systems to guide the necessary adaptions to health care to shift the focus of inquiry from the patient to health system [40]. These include promoting productive interactions, addressing the organisational of health care and embracing a community- level ecological perspective. In another example Koh’s argues: “To become health literate, organizations can begin by conducting organizational self-assessments to identify health literacy-based barriers, training staff in clear communication techniques, securing language assistance for speakers of languages other than English, and providing needed assistance to consumers while being careful not to stigmatize them [41].” The next step in organisational health literacy research needs to move from the design of tools, guides and frameworks to design, implement and evaluate organisational health literacy interventions.

A rapid realist review by Willis et al provides some important steps towards this research agenda because it examined how organisational capacity may be improved for delivering health literacy services [42]. Strategies associated with improved organisational capacity were classified into three domains including government action; organisation and practitioner action and partnership action. Government action may include strategies such as setting standards for education, reinforcing social norms through policy, measuring health literacy levels and conducting research. Organisation and practitioner action involves leadership both high level and distributed. The innovative partnership component includes working with the media, community organisations and other organisations such as schools. Willis argues that the mechanisms for change at these levels include strategies such as generating momentum, increased visibility and recognition of health literacy efforts, reducing the gap between vision and action and creating a sense of ownership for health literacy data and creating a common language and understanding [42]. The review described where capacity needs to be built but did not focus on *how* to generate change.

### Limitations

It is difficult to generalise the findings of this systematic review because only seven studies met the selection criteria. However, the included studies have high face validity in the context of organisational change. The limited number of organisation health literacy interventions in the literature may be due to the result of publication bias. Intervention research and associated strategies difficult to implement, and unsuccessful interventions are difficult to publish. The review did not include the grey literature where many organisational health literacy intervention may be reported.

### Next steps

The next step in the research on organisational health literacy needs to focus on what works in improving organisational health literacy. We do not need more tools and measures, rather we need interventions. This may be supported by a program of research to design, implement and evaluate effective interventions for building organisational health literacy. This was recommended by Willis who argues that government initiated intervention and polices are powerful strategies by which organisational capacity to improve health literacy may be affected [43].

A program of research would need to rely upon a research practice partnership that is evident in the knowledge translation cycle, defined by the Canadian Institutes of Health Research as ‘the exchange, synthesis and ethically sound application of research findings within a complex set of interactions among researchers and knowledge users’ [44].

## Conclusion

Our systematic literature review identified a gap in the research. There were no comprehensive organisational health literacy interventions in the peer reviewed literature that demonstrated change in organisational health literacy. To effect improvements in organisational health literacy, we need health systems and organisations to change. The may be best achieved by practice and research partnerships.

## Supporting information

S1 Appendix Keywords and MeSH terms used in database search. (PDF)

S2 Appendix Study selection criteria. (PDF)

S3 Appendix Methods used in the included studies. (PDF)

S4 Appendix PRISMA 2009 checklist_PLoS One. (DOC)
