# Supplementary material for: A paucity of strategies for developing health literate organisations: A systematic review
Source: PLoS One. 2018 Apr 11;13(4):e0195018. doi: 10.1371/journal.pone.0195018 (PMC5895007; doi:10.1371/journal.pone.0195018)
Supplement: S1 Appendix — (PDF) [file pone.0195018.s001.pdf]

S1 Appendix - Keywords and MeSH terms used in database search

MedLine	EMBASE	PsycINFO	CINAHL
(Organisational Change Proxy Terms)			
(184673)	*(797426)*	*(191620)*	*(106429)*
organization* health litera*.tw.	organi?ation* health litera*.tw.	organization* health litera*.tw.	TX organi?ation* health litera*
organizational commitment.mp.	organi?ational commitment.mp.	organizational commitment/	"organi?ational commitment"
"organization and administration"/	"organization and management"/	"organization and administration"/	(MH "Management")
capacity building/	capacity building/	personnel training/ or management training/	"capacity building"
"constitution and bylaws"/	organization/	"constitution and bylaws"/	(MH "Organizational Policies")
decision making, organizational/	organization/	decision making/ or group decision making/ or management decision making/	(MH "Decision Making, Organizational")
organizational culture/	organization/	organizational learning/ or organizational objectives/ or organizational climate/	(MH "Organizational Culture")
organizational innovation/	organization/	innovation/	(MH "Organizational Change")
efficiency, organizational/	organizational efficiency/	organizational effectiveness/	(MH "Organizational Efficiency")
knowledge management/	knowledge management/	knowledge management/	(MH "Knowledge Management")
personnel management/	personnel management/ or health care personnel management/	Human Resource Management/	(MH "Personnel Management")
program development/	program development/	program development/	(MH "Program Development")
public health administration/	public health service/	health care administration/	(MH "Health Services Administration")

MedLine	EMBASE	PsycINFO	CINAHL
			OR (MH "Public Health Administration")
Professional-Patient Relations/	Human relation/	therapeutic processes/ or psychotherapeutic processes/	(MH "Professional-Patient Relations")
models, organizational/	models, organizational/	organizational structure/	"organi?ational models"
organizational infrastructure.mp. organisational infrastructure.mp.	organi?ational infrastructure.mp.	organizational structure/	(MH "Organizational Structure")
health care systems.mp.	health care system/	health care systems.mp.	"health care systems"
Health Care Reform/	health care policy/	Health Care Policy/ or Health Care Reform/	(MH "Health Care Reform")
Culturally Competent Care/	transcultural care/	cultural sensitivity/ or cultural* competen*.mp.	(MH "Cultural Competence")
(Health Literacy Proxy Terms)			
(153143)	*(371846)*	*(52955)*	*(113388)*
Health Services Accessibility/	Health care delivery/	health care utilization/	(MH "Health Services Accessibility")
Self Care/	Self Care/	self care.mp.	(MH "Self Care")
information seeking behavior/	information seeking/	information seeking/	(MH "Information Seeking Behavior")
teach-back communication/	interpersonal communication/	teach-back.mp.	"teach-back"
Health Knowledge, Attitudes, Practice/	attitude to health/	health behavior/ or health attitudes/ or health knowledge	(MH "Health Knowledge") OR (MH "Attitude to Health") OR (MH "Health Behavior")
(Health Literacy Focus Term)			
(1518)	*(1671)*	*(1180)*	*(2237)*
*Health Literacy/	*Health Literacy/	*Health Literacy/	health literacy

MedLine	EMBASE	PsycINFO	CINAHL
with Limitations			
**= 363**	**= 459**	**=309**	**=24†**
