# Supplementary material for: A paucity of strategies for developing health literate organisations: A systematic review
Source: PLoS One. 2018 Apr 11;13(4):e0195018. doi: 10.1371/journal.pone.0195018 (PMC5895007; doi:10.1371/journal.pone.0195018)
Supplement: S2 Appendix — (PDF) [file pone.0195018.s002.pdf]

S2 Appendix: Study selection criteria

	Inclusion	Exclusion
**Publication language**	English	Non-English
**Publication date**	Published between January 2008 to July 2015	Published before January 2008
**Place of study**	OECD countries	Non-OECD countries
**Study type**	An intervention study (experimental or quasi-experimental trials), program evaluation or needs assessment	Non-empirical studies. Mainly descriptive studies lacking experimental or evaluative component
**Participants**	Patients: Adults, aged ≥18 years; Providers: Health care professionals in any organisational setting in health such as pharmacies, hospitals, GP and community clinics	Patients: Children aged <18 years; Providers: Specialist services such as dental and oral health services, emergency or intensive care services, care relating to eating disorders, several mental health care, intellectual or learning disability, addiction or substance abuse, and organ or tissue donations
**Interventio n**	The intervention is based at the organisation or system level, with aim to effect lasting change in organisational practice or culture from enhancing existing programs to fulfilling unmet need. Originates from and implemented within healthcare services.	The intervention mainly focuses on individual level health literacy without attempting to effect organisational change in process, policies, or practice. Originates from and implemented outside of healthcare services (e.g. higher education curriculum on health literacy).
**HMD's Framework**	Interventions is in line with the Health and Medicine Division's framework for a health literate organisation (13)	The intervention does not meet the criteria of Health and Medicine Division's framework for a health literate organisation (13)
