# Supplementary material for: A paucity of strategies for developing health literate organisations: A systematic review
Source: PLoS One. 2018 Apr 11;13(4):e0195018. doi: 10.1371/journal.pone.0195018 (PMC5895007; doi:10.1371/journal.pone.0195018)
Supplement: S3 Appendix — (PDF) [file pone.0195018.s003.pdf]

**S3 Appendix: Methods used in the included studies**

| First author, year | Type of study | Methodology | Setting | Quality rating score |
|---|---|---|---|---|
| Blake 2010 | Quasi-experimental | Qualitative: Eight focus groups with patients (four at the intervention site and four at the control site) and seven face-to-face interviews with pharmacists (four at the intervention site and three at the control site). These were conducted one month and six months after the beginning of the intervention. | Three outpatient pharmacies of an inner-city health system in a disadvantaged area of the US. A general patient pharmacy, a senior health care pharmacy and a refill pharmacy. A community pharmacy served as the control site. | Moderate |
| Shoemaker 2013 | Descriptive | Qualitative: Eight case studies guided by Rogers' Diffusion of Innovations model. Data were collected through interviews, site visit observations and the review of documents. Recruited five pharmacies that were prepared to implement the health literacy tools. A further two pharmacies who had decided not to use the tools were recruited along with a pharmacy that spontaneously decided to use the tools. | The pharmacies were diverse in chain types, organisational capacity, region and location, and patient populations served and were in the US. | High |
| Smith 2010 | Exploratory | Qualitative: As part of a pilot study environmental assessments were conducted to assess the navigation of facilities, understand ability of written and oral communication, use of technology and the implementation of policies and procedures from the view of the provider.<br>The review was completed through interviews with facility personnel and | The two settings reviewed were a stroke unit in a local freestanding rehabilitation facility and a senior independent living facility in the US. The rehabilitation facility had 60 beds and the stroke unit was housed in one wing and typically served 20 clients. The senior independent | Moderate |

| First author, year | Type of study | Methodology | Setting | Quality rating score |
|---|---|---|---|---|
| | | administrators, examination of printed material and observations using the statements from the Rudd and Anderson Health Literacy Environment Review as a guide.<br>?Navigation was assessed using a check list giving a score from 1 to 3 was used. 1 - not done; 2 – this is done, but needs improvements, 3 – this is done well. The higher the final score the better the level of health literacy.<br>Written communication was assessed using the Fry Readability Graph. Steps include 1. examining three passages of 100 words; 2. counting the number of sentences, 3 counting the total number of syllables in each text passage; 4 determining the average number of syllables and sentences among three passages. In the final step the averages are then plotted on the Fry Graph to determine the reading grade level. | living facility is a not for profit corporation. | |
| Weaver 2012 | Descriptive | Qualitative: The environmental assessment was conducted via the development of three tools which included observational assessment, key informant interviews with clinic staff and key informant interviews with patients. This was conducted by three members of the academic team. The observational assessment included | US - Primary health care - Three clinics of a federally qualified health centre in rural Missouri. The population living in the communities where the clinics were located on average had higher rates of chronic diseases, preterm and low birth-weight babies, those living below the poverty line, and uninsured residents | High |

| First author, year | Type of study | Methodology | Setting | Quality rating score |
|---|---|---|---|---|
| | | environmental indicators of health literacy practices and definitions of those indicators according to each domain. Similarly, to Smith the responses were captured using categories of yes/no and 1 - not done; 2 – this is done, but needs improvements, 3 – this is done well.<br>The patients and staff interviews contained approximately 20 open-ended questions. | compared to the rest of the State. | |
| Cawthon 2014 | Quasi experimental | Mixed methods: The uptake of the Brief Health Literacy Screen (BHLS) was electronically monitored through an established quality and safety monitoring system software. The system enabled tracking of health literacy item completion over time and by nursing unit.<br>The hospitals electronic health record (EHR) was modified to incorporate the BHLS. Clinical and administrative data from the EHR were maintained in the Enterprise Data Warehouse (EDW) that was later queried for analysis.<br>Three methods were used to collect data: querying the EDW; direct observations; and focus groups, interviews and process recordings. | The Brief Health Literacy Screen was implemented in a University Medical Centre in Nashville, Tennessee. This included a 658-bed hospital, outpatient facilities that receive more than 1.5 million visits each year. The setting for the research included all inpatient adult units, the adult emergency department and three adult primary care practices. | Moderate |
| Johnson 2014 | Descriptive | Qualitative methods were used for the environmental assessment. The focus was on addressing health | Rural hospital in South Australia | Moderate |

| First author, year | Type of study | Methodology | Setting | Quality rating score |
|---|---|---|---|---|
| | | literacy from the view of the patient. One patient was the reviewer for the phone call, another for the web page and both patients undertook the walking interview and provided feedback and suggested improvements to the navigation of the health service. The Walking Interview Guide was used for the navigation assessment. The Safety and Quality Coordinator was the guide for the walking interview. | | |
| Groene and Rudd 2010 | Descriptive | Mixed methods were used for the environmental assessment. The Walking Interview Guide was used for the navigation assessment.<br>Written communication as assessed using the Flesch-Szigriszt readability formula which is based on the length of words and sentences. The different levels of text difficulty are scored from 1 to 100 and include the equivalent education requirements. Materials scored at less than 40 points are rated as very difficult generally associated with skills acquired with a university degree. Materials scoring greater than 80 are rated as very easy and are generally associated with skills at a primary school level.<br>Oral communication was assessed using a nurse administered survey after the cataract surgery follow | Ten hospitals in Catalonia, Spain<br>These hospitals served a population close to 3 million people.<br>Participating hospitals were members of the Catalan Network of Health Promoting Hospitals. This is a network which seeks to improve services for vulnerable populations as well as offer a strong orientation toward health promotion, patient centeredness, community involvement and environmental sustainability.<br>The focus of attention for the health literacy assessment was cataract surgery in outpatient departments. | High |

| First author, year | Type of study | Methodology | Setting | Quality rating score |
|---|---|---|---|---|
| | | up visit. There were 21 items related to the overall accessibility of patient materials and the opportunity to ask questions and the clarity of the explanations provided. Answers were either dichotomous or included an assessment scale with values from 1 to 10.<br>Feasibility was assessed using a semi-structured questionnaire with hospital coordinators. Questions included the value of the exercise and suggestions for improving the process. | | |
